# Mortality after Distal Radius Fracture in Men and Women Aged 50 Years and Older in Southern Norway

**DOI:** 10.1371/journal.pone.0112098

**Published:** 2014-11-07

**Authors:** Jannike Øyen, Andreas P. Diamantopoulos, Glenn Haugeberg

**Affiliations:** 1 National Institute of Nutrition and Seafood Research, Bergen, Norway; 2 Department of Rheumatology, Hospital of Southern Norway, Kristiansand, Norway; 3 Department of Neurosciences, Norwegian University of Science and Technology, Trondheim, Norway; David Geffen School of Medicine, United States of America

## Abstract

Increased mortality rates in patients sustaining hip and vertebral fractures are well documented; however in distal radius fracture patients the results are conflicting. The aim of this study was to examine short- and long-term mortality in distal radius fracture patient in comparison with the background population. Patients aged ≥50 years with distal radius fracture living in Southern Norway who suffered a fracture in the two year period 2004 and 2005 were included in the study. The mortality risk of the standard Norwegian population was used to calculate the standardized mortality ratio (SMR). The number of distal radius fractures was 883 (166 men and 717 women). Mean age was 69 years (men 65 years and women 70 years). After one year the overall mortality rate was 3.4% (men 5.4% and women 2.9%) and after five years 4.6% (men 4.0% and women 4.8%). The SMR for men and women compared to the Norwegian population for the first year was 1.6 (95% confidence interval (CI): 0.6, 2.7) and 0.9 (95% CI: 0.4, 1.2), respectively, and after five years 1.7 (95% CI: 0.3, 3.0) and 2.0 (95% CI: 1.2, 2.7). Stratified on age groups (50–70 and >70 years) an increased SMR was only seen in female patients aged >70 years five years after the fracture (SMR: 1.9, 95% CI: 1.1, 2.6). In conclusion, increased SMR was found in female patients aged >70 years five years after the distal radius fracture, but not in men or in women younger than 70 years.

## Introduction

Distal radius fracture is one of the most common osteoporotic fractures in middle-aged and older men and women [1]. The incidence of distal radius fractures has been shown to be higher in Norway than in other European countries, including other Scandinavian countries [2], [3], [4], [5]. Fragility distal radius fracture which occurs with mean age ∼70 years has been shown to be a predictor of future more severe fractures e.g. hip fracture which occurs with mean age ∼80 years [6].

Increased mortality rates in patients sustaining hip and vertebral fractures are well documented [7], [8], [9], [10], however, in distal radius fracture patients conflicting results have been reported [7], [11], [12], [13], [14]. Increased mortality [12], increased survival [13], as well as no differences in mortality rate data [7], [11], [14] has been published. This may be explained by differences in study design, or more likely differences in frailty of the distal radius fracture in patients studied. Despite the high incidence of distal radius fracture in Norway, the mortality has not been examined. Information on a possible increased mortality risk in all or in subsets of distal radius fracture patients may thus be important to develop a targeted strategy to improve clinical outcome.

Thus, the aim of this study was to evaluate the short- and long-term mortality after distal radius fracture in males and females in Southern Norway, and to compare it with the background Norwegian population.

## Methods

### Ethical approval

The study was approved by the Regional Committee for Medical Research Ethics for Southern and Eastern Norway.

### Distal radius fracture patients

Annual incidence data on distal radius fracture from Southern Norway in individuals aged 50 years and older has previously been reported for the two year period 2004 and 2005 [3]. As previously described in details [3], the patients were recruited from the four hospitals in Southern Norway, located in Kristiansand, Arendal, Flekkefjord, and Mandal (Vest- and Aust-Agder counties). These hospitals are the only referral centers for orthopedic trauma in Southern Norway. The hospital electronic diagnosis registers were used to identify all distal radius fracture patients coded S52.5 (lower end of radius), S52.6 (lower end of radius and ulna), and S62.8 (other and unspecified parts of wrist and hand) according to the International Classification of Diseases 10^th^ Revision (ICD-10) in the two year recruitment period. For all individuals diagnosed with a distal radius fracture, data on sex, birth, and place of residency were collected. Further, we examined the patients' medical- and X-ray records to confirm the distal radius fracture diagnosis. As previously described, a high- or a low-energy fracture was determined based on information when reviewing the medical records [3]. In short, a low-energy fracture was defined as a result of falling from standing height or less, while high-energy fracture was defined as any other type of trauma (e.g. falling from height higher than standing height and motor vehicle accident) [15].

The follow-up time for the distal radius fracture patients was from the month when the fracture occurred to death, or to the censoring dates of January 1^st^, 2009 and 2010.

### Statistical methods

Distal radius fracture mortality rates were calculated for the first and the fifth year, for all, separately for each gender, and for the age groups 50–70 and >70 years. The 70 years cutoff was chosen as it was close to the mean age of our patients. To calculate the standardized mortality ratio (SMR), the mortality risk of the standard Norwegian population was used [16], for all fracture patients and for those with a low-energy fracture separately. All analyses were performed using the SPSS version 17.0 (SPSS, Chicago, IL, USA). Statistical significance was defined as p<0.05.

## Results

As previously reported [3], the number of individuals (≥50 years old) with distal radius fractures in the geographic area of Southern Norway in the two years period 2004 and 2005 was 883 (166 men and 717 women) and for low- and high-energy fracture separately 799 (118 men and 681 women) and 84 (48 men and 36 women), respectively. Mean age for all included patients was 69 years (men 65 years and women 70 years) and for low- and high-energy fracture patients separately 70 years (men 67 years and women 71) and 64 years (men 62 years and women 66).

Data on mortality rates and SMR are given in table 1. After one year the overall mortality rate was 3.4% (men 5.4% and women 2.9%), and after five years 4.6% (men 4.0% and women 4.8%). The SMR for men and women compared to the Norwegian population after one year was 1.6 (95% confidence interval (CI): 0.6, 2.4) and 0.9 (95% CI: 0.4, 1.2), respectively. After five years the mortality rates were significantly higher among female distal radius fracture patients compared to the general population (SMR: 2.0, 95% CI: 1.3, 2.7). In males, no significant increased mortality rates were observed (Table 1).

**Table 1:** Percentage mortality rates and standardized mortality ratios (SMR) in distal radius fracture patients one and five years after the fracture.

	All (n = 883)	Males (n = 166)		Females (n = 717)	
	Mortality rates (%)	Mortality rates (%)	SMR (95% CI)	Mortality rates (%)	SMR (95% CI)
After 1 year					
All	3.4	5.4	1.6 (0.6, 2.4)	2.9	0.9 (0.4, 1.2)
50–70 years	0.8	0.9	1.1 (−1.1, 3.3)	0.8	1.4 (0.3, 3.1)
>70 years	6.0	13.3	1.7 (0.5, 2.9)	4.8	0.8 (0.4, 1.2)
After 5 year					
All	4.6	4.0	1.7 (0.3, 3.0)	4.8	2.0 (1.3, 2.7)
50–70 years	1.4	0.9	1.3 (−0.2, 3.9)	1.5	2.7 (0.3, 5.1)
>70 years	8.9	10.8	1.7 (0.2, 3.3)	8.6	1.9 (1.1, 2.6)

Data on mortality rates and SMR by sex and age groups are presented in table 1. For the age group 50–70 years (453 patients, males 106, females 347) the overall mortality rates were 0.8% after one year and 1.4% after five years, whereas for the age group >70 years (430, males 60, females 370) the overall mortality rates were 6.0% after one year and 8.9% after five years. Male patients aged >70 years had a significantly higher mortality rate than females one year after the fracture (13.3% versus 4.8%, p<0.01), but not after five years. The SMR for the age group 50–70 years was not significant in any of the groups (Table 1). However, for female distal radius fracture patients in the age group >70 years the SMR was significant higher five years after the fracture compared to the Norwegian population in general (SMR: 1.9, 95% CI: 1.1, 2.6) (Table 1).

The results did not substantially change when only low-energy distal radius fractures where included in the analyses as shown in table 2.

**Table 2:** Percentage mortality rates and standardized mortality ratios (SMR) in low-energy distal radius fracture patients one and five years after the fracture.

	All (n = 799)	Males (n = 118)		Females (n = 681)	
	Mortality rates (%)	Mortality rates (%)	SMR (95% CI)	Mortality rates (%)	SMR (95% CI)
After 1 year					
All	3.3	5.9	1.8 (0.5, 2.7)	2.9	0.9 (0.5, 1.3)
50–70 years	1.0	1.4	1.6 (−1.5, 4.7)	0.9	1.5 (−0.2, 3.2)
>70 years	5.6	12.0	1.6 (0.4, 2.8)	5.0	0.8 (0.4, 1.2)
After 5 year					
All	4.7	5.7	1.7 (0.3, 3.1)	5.1	2.0 (1.3, 2.7)
50–70 years	1.5	1.5	1.9 (−1.8, 5.6)	1.6	2.9 (0.2, 5.4)
>70 years	9.4	13.5	1.9 (0.1, 3.7)	8.9	1.9 (1.3, 2.5)

## Discussion

In this study compared to the general population increased SMR after five years follow-up was only found in female distal radius fracture patients aged >70 years and was not found in men or in women younger than 70 years. One year follow-up did not show any significant elevated mortality rates either for men or for women with distal radius fracture.

Our one year follow-up mortality data are in accordance with Endres et al. [14] who observed that 3% of the distal radius fracture patients from Germany died during short-term follow-up (1.5 year). However, our mortality rates are lower than those Johnell et al. [7] observed at one (6%) and five years follow-up (26%) in a Swedish study. Rozental et al. [12] reported that 21% of the distal radius fracture patients from US (29% of men and 19% of women) died during a follow-up period of seven years. The aforementioned higher mortality rates may be explained by that their follow-up period was two years longer and the patients were in mean eight years older than in our study, thus the frailty might have been increased in these patients compared to our patients.

Most of the studies investigating mortality in distal radius fracture patients have not detected any differences compared to the general population [7], [11], [14], which is in accordance with our findings for men and one year follow-up in women. In the study by Rozental et al. [12], patients aged 65 years and older were included and followed for seven years, and increased mortality in both men and women were found [12]. Interestingly, when we stratified on age groups, female patients at increased mortality risk were older than 70 years, whereas women younger than 70 years did not have an increased mortality. Our results thus indicate that distal radius fracture may be a marker of increased risk of future death in older women. However, the previous reported data are conflicting. In the study by Shortt et al. [13] recruiting patients from a single trauma unit they even observed a higher survival among distal radius fracture patients compared to age matched controls. The median age was similar to our included patients and a slightly increased survival was observed in all age groups up to 10 years of follow-up [13].

The conflicting findings may be explained by different study design, unlike definition of distal radius fracture and thus inclusion of patients, as well as differences in frailty or comorbidities, and thus the risk of fall. Unfortunately, we did not have any information on frailty, previous falls, comorbidities and other risk factors associated with mortality, or causes of death. Therefore, our study does not identify whether or not distal radius fracture is an independent risk factor in middle-aged and older women five years after the fracture. Rozental et al. [12] presented data on comorbidity, and although the mortality rates were higher among males no significant associations with any of comorbidities were observed. Shortt et al. [13] argued that individuals surviving the first year after the fracture may be physiologically more robust and have similar or even better survival rates compared to the general population. This hypothesis may also be partially supported by our data as the mortality risk in our patients older than 70 years was significantly higher in men than in women after one year but not after five years.

Our study has limitations. The numbers of distal radius fractures may have been underestimated due to the retrospective study design as the diagnosis system may not have identified all distal radius fracture patients due to wrong ICD-10 coding. Nonetheless, a retrospective study design may be superior to a prospective one. We have previously in a hip fracture study shown that more patients were identified retrospectively using the ICD-10 diagnosis code system compared to when patients were identified prospectively [17].

Previous data have showed a high prevalence of low bone mineral density [18], [19], [20], [21] and low vitamin D levels [22] in distal radius fracture patients. Taking into account the above and the increased mortality in older women five year after the fracture, more efforts should be placed into identifying risk factors in patients after their low-energy distal radius fracture.

In conclusion, increased SMR was found in female patients aged >70 years five years after the distal radius fracture, but not in men or in women younger than 70 years. Distal radius fracture in older women may thus be a clinical marker of poor future outcome and health.
